# Identification and Spatiotemporal Expression of Adenosine Deaminases Acting on RNA (ADAR) during Earthworm Regeneration: Its Possible Implication in Muscle Redifferentiation

**DOI:** 10.3390/biology9120448

**Published:** 2020-12-05

**Authors:** Yoo Bin Yoon, Yun-Sang Yu, Beom Jun Park, Sung-Jin Cho, Soon Cheol Park

**Affiliations:** 1 Department of Life Science, Chung-Ang University, Seoul 06974, Korea; yoobin201@cau.ac.kr (Y.B.Y.); dkehfzmfltm@cau.ac.kr (B.J.P.); 2 School of Biological Sciences, College of Natural Sciences, Chungbuk National University, Cheongju 28644, Korea; yys9002@chungbuk.ac.kr

**Keywords:** earthworm, regeneration, RNA editing, ADAR, muscle redifferentiation

## Abstract

**Simple Summary:**

Among the animal species capable of regenerating missing body parts, a species of earthworm, *Perionyx excavatus*, has the most powerful regeneration capacity, which can completely and regenerate an amputated head and tail. Earthworm regeneration is a form of epimorphosis, a simple mode of development in adults that occurs around the sites of damage rather than throughout the body. In order to achieve this process, the earthworm must have molecular tools via which a variety of cell and tissue types can be precisely recovered from the pluripotent (or possibly totipotent) blastemal cells. Adenosine to inosine (A-to-I) RNA editing catalyzed by adenosine deaminases acting on RNA (ADAR) can generate substantial transcriptome and proteome variability and provide an ideal tool for cell and tissue re-specification. To understand the role of ADAR during earthworm regeneration, the molecular characteristics of an *ADAR* gene identified from *P. excavatus* (*Pex-ADAR*) were analyzed, and its spatial and temporal expression patterns were observed during regeneration. Domain analysis showed that *Pex-ADAR* is a member of the ADAR1 class. Its expression level primarily increases when and where muscle redifferentiation is actively taking place, suggesting that the RNA-editing enzyme *Pex-ADAR* is involved in muscle redifferentiation.

**Abstract:**

Adenosine deaminases acting on RNA (ADAR) catalyze the hydrolytic deamination of adenosine (A) to produce inosine (I) in double-stranded RNA substrates. A-to-I RNA editing has increasingly broad physiological significance in development, carcinogenesis, and environmental adaptation. *Perionyx excavatus* is an earthworm with potent regenerative potential; it can regenerate the head and tail and is an advantageous model system to investigate the molecular mechanisms of regeneration. During RNA sequencing analysis of *P. excavatus* regenerates, we identified an *ADAR* homolog (*Pex-ADAR*), which led us to examine its spatial and temporal expression to comprehend how *Pex-ADAR* is linked to regeneration. At first, in domain analysis, we discovered that *Pex-ADAR* only has one double-stranded RNA-binding domain (dsRBD) and a deaminase domain without a Z-DNA-binding domain (ZBD). In addition, a comparison of the core deaminase domains of *Pex-ADAR* with those of other ADAR family members indicated that *Pex-ADAR* comprises the conserved three active-site motifs and a glutamate residue for catalytic activity. *Pex-ADAR* also shares 11 conserved residues, a characteristic of ADAR1, supporting that *Pex-ADAR* is a member of ADAR1 class. Its temporal expression was remarkably low in the early stages of regeneration before suddenly increasing at 10 days post amputation (dpa) when diverse cell types and tissues were being regenerated. In situ hybridization of *Pex-ADAR* messenger RNA (mRNA) indicated that the main expression was observed in regenerating muscle layers and related connective tissues. Taken together, the present results demonstrate that an RNA-editing enzyme, *Pex-ADAR*, is implicated in muscle redifferentiation during earthworm regeneration.

## 1. Introduction

RNA editing, a post-transcriptional modification of RNA molecules, is a widespread phenomenon in all metazoans. The most common RNA editing event is the chemical conversion of adenosine to inosine (A-to-I) catalyzed by adenosine deaminases acting on RNA (ADAR) enzymes, which mainly act on double-stranded RNA (dsRNA) substrates. This base modification in RNA molecules confers substantial transcriptome and proteome variability and impacts protein function, microRNA (miRNA) biogenesis or target spectrum, and RNA fate (transport, splicing), and it is thought to be associated with mammalian brain and neural crest cell development [1,2], carcinogenesis [3,4], genetic disorder [5], and environmental adaptation [6,7]. To carry out this enzyme activity, all ADARs share a common domain architecture consisting of at least one N-terminal dsRNA-binding domain (dsRBD) and a C-terminal deaminase domain [8].

The first *ADAR* gene to be identified was *ADAR1* from oocytes of vertebrate *Xenopus laevis* [9]. Subsequently, *ADAR2* was described, which is critically required for *GluR-B* Q/R editing, and several other editing events that largely affect protein recoding [10,11]. Through genome analysis of 14 lophotrochozoan species, some ADAR homologs were identified and classified as ADAD, ADAR1, or ADAR2 on the basis of the number of DNA- and RNA-binding domains and on the global protein architecture. According to their classification, in annelids, the genomes of *Capitella teleta* (Polychaeta) and *Helobdella robusta* (Hirudinea) are thought to possess *ADAR-like* genes, consisting of one ADAD, one ADAR, and two ADAR2 in *C. teleta* and only one ADAR2 in *H. robusta* [12]. However, in the Oligochaete, another important class of annelid that includes terrestrial earthworms, there is little accumulated information on ADAR and RNA editing.

Earthworms show a wide spectrum of regenerative potential capable of reconstructing body parts lost due to injury. Among the earthworm species, *Perionyx excavatus* has the most powerful regenerative capacity and can completely regenerate an amputated head and tail within approximately 3 weeks post amputation. In particular, during complete anterior regeneration, *P. excavatus* can reconstruct the essential body parts, including the brain, heart, and reproductive organs (i.e., testis, ovary, seminal vesicle, and clitellum) [13], and the species provides significant benefits compared with planarian and hydras for exploring regenerative mechanisms. It is generally believed that earthworm regeneration is an epimorphosis, which is characterized by the dedifferentiation of adult tissue to form a highly proliferating cell mass called a blastema, followed by its re-specification into appropriate cell types [14]. Therefore, its regeneration requires the activation of cellular plasticity in dedifferentiated blastemal cells. ADAR-catalyzed RNA editing facilitates molecular diversity and would be an ideal tool for cellular plasticity activation. It has been demonstrated that, in urodele regeneration, the expression levels of *ADAR1* and *ADAR2* messenger RNA (mRNA) are differentially expressed during different phases of regeneration in multiple tissues [15].

Through RNA sequencing analysis of the regenerates of *P. excavatus*, we identified a full-length complementary DNA (cDNA) sequence showing significant homology to mammalian ADAR1. To our knowledge, this is the first report on the molecular characterization of an earthworm (Oligochaete) ADAR and its possible implication in muscle redifferentiation during regeneration.

## 2. Materials and Methods

### 2.1. Animals and Computational Sequence Analysis

Sexually mature *P. excavatus* obtained from a commercial source (Seoul, Korea) were reared using a method previously described [16]. Before being used, the earthworms were placed in Petri dishes lined with filter paper moistened with earthworm saline for 48 h to purge the gut contents, which may interfere with cryosectioning. The open reading frame (ORF) was determined using the ORF finder on the server of National Center of Biotechnology Information (NCBI). The subcellular localization of *Pex-ADAR* was predicted by PSORT II [17].

### 2.2. Comparative and Phylogenetic Analyses

Amino-acid sequences of ADAR1 were retrieved from the GenPept Database via protein Basic Local Alignment Search Tool (BLASTP) and UniProt (http://www.uniprot.org/). Amino-acid sequence alignment was carried out using ClustalX software (http://www.clustal.org/clustal2/). Phylogenetic analysis was performed via the neighbor joining (NJ) method, using the NJplot. (http://doua.prabi.fr/software/njplot). Bootstrap analysis was performed with 1000 replications. The phylogenetic tree was built with ADAR1 orthologs of metazoan animal models (Lophotrochozoa: *Perionyx excavatus* (MT905407), *Pecten maximus* (XP_033751634), *Mizuhopecten yessoensis* (OWF54145), *Haliotis diversicolor* (A0A5B7L1W7), *Octopus vulgaris* (XP_029636176), and *Lingula anatina* (XP_013411771); Deuterostomia: *Homo sapiens* (P55265), *Rattus norvegicus* (P55266), *Mus musculus* (Q99MU3), *Gallus gallus* (A0A1D6Y7R2), *Xenopus laevis* (O12982), *Danio rerio* (E9QIW2), and *Takifugu rubripes* (H2TCA4)), ADAR2 orthologs (*Homo sapiens* (P78563), *Rattus norvegicus* (P51400), *Mus musculus* (Q91ZS8), and *Danio rerio* (Q90VY9)), and ADAR3 orthologs (*Homo sapiens* (Q9NS39), *Rattus norvegicus* (P97616), *Mus musculus* (Q9JI20), and *Danio rerio* (X2BQ53)).

### 2.3. Quantitative Real-Time PCR

Total RNA was isolated from both the head and the tail regenerates of *P. excavatus* during regeneration using TRIzol (Ambion, Austin, TX, USA) at the times indicated. We selected mRNA from total RNA using oligo (dT) primers (Promega, Madison, WI, USA) and then reverse-transcribed the mRNA into cDNA (SuperScript II First-Strand Synthesis System for RT-PCR, Invitrogen, Waltham, MA, USA). Quantitative PCR (qPCR) was performed using WizPure™ qPCR Master (SYBR) (Wizbiosolutions, Korea) with specific primer pairs on an Applied Biosystems Stepone plus real-time PCR System. The sequences of primer pairs were as follows: *Pex-ADAR* (forward) 5′–TCCGTGTGGAGATGGTTCAC–3′ and (reverse) 5′–CGCCACGAAGAATTCCATCC–3′; *Pex-GAPDH* (forward) 5′–TCGGTCGTTTGGTGATGAGA–3′ and (reverse) 5′–TTCCATCGTGGTGGACTTCA–3′. Relative quantification of mRNA was conducted using the comparative 2^−ΔΔCt^ method with glyceraldehyde 3-phosphate dehydrogenase (GAPDH) as the reference gene. All data are expressed as the mean ± the standard error of the mean (SEM) and were analyzed using GraphPad Prism 6.01 (GraphPad Software, Inc.). Differences between groups were tested by one-way ANOVA.

### 2.4. Fluorescent In Situ Hybridization

Fluorescent in situ hybridization (FISH) on cryosections was performed as previously described [18] using a riboprobe of 594 bp with 4,6-diamidino-2-phenylindole (DAPI, Sigma, Saint Louis, MO, USA) to visualize cell nuclei. Briefly, prehybridization buffer was replaced with fresh hybridization buffer (50% formamide, 5× Saline Sodium Citrate (SSC), 1× Denhardt’s solution, 0.1% 3-[(3-cholamidopropyl) dimethylammonio]-1-propanesulfonate (CHAPS), 100 mg/mL Heparin, 0.1% Tween-20, 100 mg/mL transfer RNA (tRNA)) containing 4 ng/mL of the corresponding riboprobe, and the specimens were incubated at 67 °C overnight. Washed specimens were incubated at room temperature for 1.5 h in 1% blocking reagents (Roche) in Phosphate buffered saline with Tween-20 (PBT), then incubated at 4 °C for 16 h with 1/1000 anti-digoxigenin conjugated with horse-radish peroxidase (anti-DIG/POD) antibody (Roche) in 1% blocking reagents. For FISH, we used the Tyramide Signal Amplification (TSA) Plus Kit (PerkinElmer, Wellesley, MA, USA). After washing with PBT, we preincubated the specimens in maleic acid buffer (100 mM maleic acid, 150 mM NaCl, pH 7.5) for 15 min; then, the specimens were blocked in 1% blocking reagent for nucleic acids for 2 h at room temperature and incubated at 4 °C for 16 h with 1/1000 anti-DIG/POD in 1% blocking reagent. After incubation, the specimens were rinsed twice with TNT buffer (0.1 M Tris–HCl pH 7.5, 0.15 M NaCl, 0.1% Tween-20). Subsequent washes with TNT at room temperature were followed by a single rinse with NEN TSA Plus amplification solution. The color reaction was initiated by adding a 1:100 dilution of reconstituted cyanine-3 tyramide in amplification solution. The stained specimens were dehydrated in ethanol and mounted in Fluoromount-G (Southern Biotech, Birmingham, AL, USA), and images were taken with an EVOS FL Auto2 (Invitrogen, Waltham, MA, USA).

## 3. Results

### 3.1. Sequence and Domain Analyses

**Figure 1:**
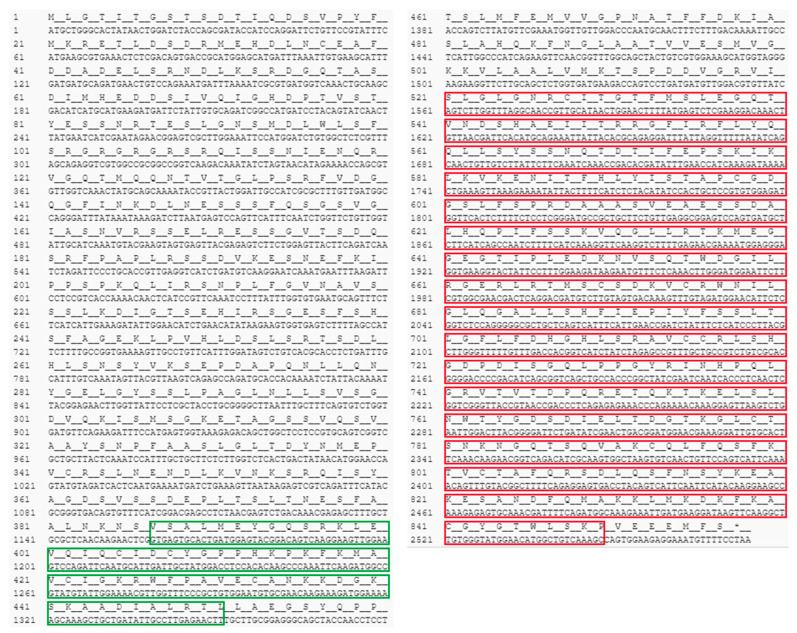
Nucleotide and deduced amino-acid sequences of earthworm adenosine deaminases acting on RNA (*ADAR*) found in the earthworm *Perionyx excavatus* (*Pex-ADAR*). The open reading frame of the *Pex-ADAR* gene consists of 2571 nucleotides encoding 857 amino acids. The double-stranded RNA-binding domain (dsRBD) and deaminase domain are presented in the green and red boxes, respectively. The stop codon is indicated by an asterisk. Residue numbers for nucleotides and amino acids are indicated in the left row. The sequence data for *Pex-ADAR* are available from the National Center for Biotechnology Information (NCBI) through accession number MT905407.

The nucleotide and predicted amino-acid sequences of *ADAR* found in the earthworm *P. excavatus* (*Pex-ADAR*) are shown in Figure 1.

**Figure 2:**
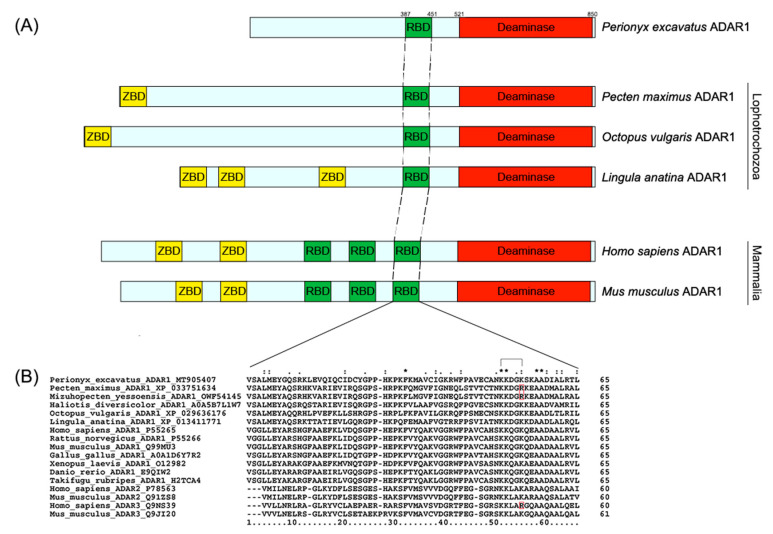
Primary structures of ADAR1s showing the arrangements of dsRBD and deaminase domains (**A**), and multiple sequence alignment of dsRBDs of ADAR family (**B**). (**A**) Protein domains are represented by the boxes and colored as follows: red, deaminase domain; green, dsRNA-binding domain; yellow, *Z*-DNA-binding domain. (**B**) All dsRBD sequences of ADAR1s are highly conserved in size and residues, encompassing a KKXXK(R) motif (X is any amino acid), which is important for dsRNA binding in this enzyme. The conservative replacement of K to R is indicated in red. The accession number of each sequence is denoted after the species name. Conserved residues are indicated with an asterisk (*), while (:) and (.) indicate conservative and semiconservative substitutions.

The ORF of *Pex-ADAR* consists of 2571 bp corresponding to a polypeptide of 857 amino-acid residues. Figure 2A exhibits the domain structure diagrams of the ADAR proteins present in mammals and lophotrochozoans. *Pex-ADAR* has only one dsRBD and a deaminase domain without a *Z*-DNA-binding domain (ZBD), and its subcellular localization was predicted to be nuclear. Because *Pex-ADAR* has a single dsRBD, we compared the sequence of the domain with those of dsRBDs at the equivalent position of diverse ADARs. All ADAR1 dsRBD sequences analyzed are highly conserved in size (65 amino acids) and residues, encompassing the KKXXK(R) motif (X is any amino acid and R indicates the conservative replacement of K), which is important for dsRNA binding in this enzyme (Figure 2B) [19].

**Figure 3:**
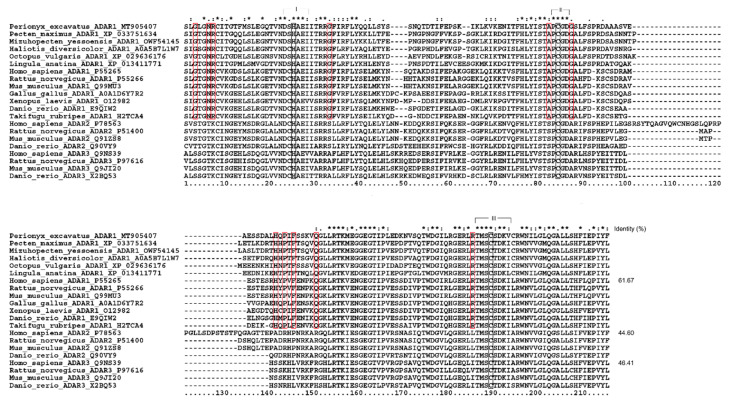
Alignment of core deaminase domains from the ADAR family, showing residues characteristic of ADAR1 proteins. The characteristic residues of ADAR1 are shown in closed red boxes. The three active site motifs are bracketed, within which zinc-binding residues (histidine or cysteines) are represented in closed black boxes. Sequence identity (%) is expressed in comparison with human ADAR1, 2, and 3.

In addition, the differences between the sequences of the deaminase core region represent a reliable index to determine the ortholog’s relationship to newly sequenced *ADARs* [20]. The alignment of core deaminase domains of *Pex-ADAR* with other ADAR family members is shown in Figure 3. Like the other ADARs, the core region of *Pex-ADAR* encompasses the three active-site motifs, including zinc-chelating residues (histidine or cysteine in black boxes) and a glutamate (E) residue in the first active-site motif for catalytic activity. It is also noted that *Pex-ADAR* shares 11 conserved residues, a characteristic of ADAR1, distinct from those of ADAR2 and 3, and the core deaminase domain of *Pex-ADAR* exhibits 61.7% sequence identity with hADAR1, while less than 50% for hADAR2 and hADAR3. Furthermore, phylogenetic analysis revealed that *Pex-ADAR* can be clustered with ADAR1s from other lophotrochozoans, including mollusks, which were grouped separately from those of deuterostomes (Figure S1, Supplementary Materials). These facts support the possibility that *Pex-ADAR* is a member of the ADAR1 class.

### 3.2. Temporal Expression of Pex-ADAR mRNA during Regeneration

**Figure 4:**
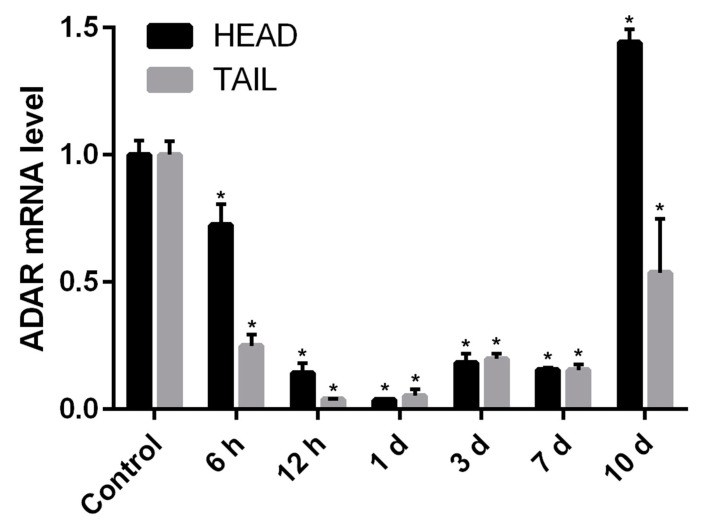
Temporal expression analysis of *Pex-ADAR* messenger RNA (mRNA) using real-time qPCR during head and tail regeneration of *P. excavatus*. In both the regenerating head and the regenerating tail, *Pex-ADAR* mRNA expression began to decrease before 6 h post amputation, showing the minimal expression around 24 h. After that time, *Pex-ADAR* transcription gradually rebounded, before suddenly increasing at 10 days post amputation (dpa), especially in the regenerating head tissue. The relative level was normalized to glyceraldehyde 3-phosphate dehydrogenase (GAPDH). The data, obtained from three independent experiments, are expressed as the mean ± the standard error of the mean (SEM). An asterisk indicates statistical significance (*p* < 0.01) compared with unamputated control.

The expression level of *Pex-ADAR* mRNA during the head and tail regeneration of *P. excavatus* was determined using qPCR. During the regeneration of both parts, *Pex-ADAR* mRNA expression began to remarkably decrease before 6 h post amputation, showing the minimal expression around 24 h post amputation. After this time, *Pex-ADAR* expression gradually rebounded and then suddenly increased at 10 days post amputation (dpa), especially in the regenerating tissue of the head (Figure 4).

### 3.3. Spatial Expression of Pex-ADAR mRNA during Regeneration

**Figure 5:**
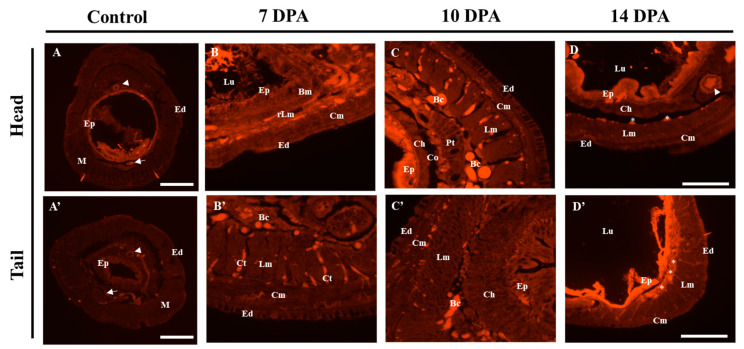
Spatial expression analysis of *Pex-ADAR* mRNA using fluorescent in situ hybridization (FISH) on cryosections during head and tail regeneration of *P. excavatus*. (**A**,**A’**) In the unamputated control, most of the *Pex-ADAR* mRNA expression signaling was observed in the gut epithelia (Ep) (×40, scale bar: 650 µm). (**B**,**B’**: 7 dpa; **C**,**C’**: 10 dpa; **D**,**D’**: 14 dpa) In the regeneration of both head and tail, *Pex-ADAR* mRNA expression was primarily found in muscle layers and around connective tissues (×200, scale bar: 125 µm). Arrow, ventral nerve cord; arrow head, dorsal vessel; Bm, basement membrane; Bc, blastemal cell; Ch, chloragog tissue; Cm, circular muscle; Co, coelom; Ct, connective tissue; Ed, epidermis; Ep, epithelial cells; Lm, longitudinal muscle; Lu, lumen of gut; M, muscle layer; Pt, peritoneum; rLm, reconstructing longitudinal muscle; T, typhlosole.

In the intact control, the majority of *Pex-ADAR* mRNA expression was observed in the gut epithelia, with minor expression in the ventral nerve cord and dorsal vessel. No positive signal was observed in the chloragogen, the muscular, and the epidermal layers (Figure 5A,A’ and Figure S2A, Supplementary Materials). At 7 dpa, after *Pex-ADAR* expression was rebounded at 3 dpa, we observed its expression in the reconstructing longitudinal muscle region and the gut epithelial cells of the head (Figure 5B). At the same period, in the regenerating tail, positive expression signals were detected primarily in the connective tissues between the longitudinal muscle bundles and in blastemal cells (Bc) on the coelomic end of the longitudinal muscle layer (Figure 5B’).

At 10 dpa, when the boundaries between each tissue layer became clearer, the major signals in the head came from the gut epithelium and blastemal cells between the longitudinal muscle layer and the peritoneum (Pt) (Figure 5C and Figure S2B, Supplementary Materials). During the regeneration of the tail, expression mainly appeared at the blastemal cells inside the coelom and gut epithelium (Figure 5C’ and Figure S2B, Supplementary Materials). In addition, weak signals were seen in the circular muscle layer in both regenerations (Figure 5C,C’). On 14 dpa, when the reorganization of the major tissues had somewhat advanced, the spatial expression of *Pex-ADAR* mRNA was similar to that of the control, with expression at the coelomic end of the longitudinal muscle layer (white stars in Figure 5D,D’).

## 4. Discussion

All metazoan ADAR proteins share a common domain architecture consisting of two or three N-terminal dsRBDs and a C-terminal catalytic deaminase domain that often has N-terminal ZBDs [8]. However, several ADARs have been reported that do not include ZBDs [21]. The ZBD of ADAR1 is the first domain discovered to bind *Z*-DNA with high affinity and probably functions to facilitate the binding of ADAR1 to transcriptionally active sites by localizing this enzyme to sites of active transcription [22,23]. However, deletion variants of ADAR1 lacking the ZBD are likely to interact normally with chromosomes and are enzymatically active [23], indicating that ZBDs are not essential and contribute only marginally to chromosomal targeting of the enzyme [24]. In many cases, ADARs contain two or more dsRBDs, and the duplication or triplication of the dsRBD confers a higher affinity for dsRNA [25]. The number of dsRBDs contributes to the substrate specificity of the enzyme [26]. As an example, ADR-2 of *Caenorhabditis elegans* with a single dsRBD displays RNA editing activity and has a distinct role from ADR-1, which has two dsRBDs [27].

ADAR family proteins are classified into ADAD, ADAR1, and ADAR2 on the basis of the number of DNA- and RNA-binding domains: ADAD has one RNA-binding domain, ADAR2 has two RNA-binding domains, and ADAR1 has at least one DNA- and one RNA-binding domain [12,28]. In line with this categorization, *Pex-ADAR* belongs to a class of ADADs comprising a single dsRBD and a deaminase domain. However, the sequence difference in the deaminase core region between ADARs, particularly close to the catalytic site, enables us to assign newly sequenced ADARs to corresponding orthologs [20]. A comparison of the deaminase core region of *Pex-ADAR* with that of other ADAR1 proteins shows the conservation of three active-site motifs and a glutamate (E) residue for catalytic activity. Additionally, in phylogenetic analysis, *Pex-ADAR* was grouped together with another metazoan ADAR1. These findings strongly support that *Pex-ADAR* is a member of the ADAR1 family.

Very recently, the morphological processes involved in *P. excavatus* head regeneration were reported, mainly focusing on nerve regeneration. Its head regeneration primarily accompanies wound healing and blastemal cell proliferation at 1–3 dpa, followed by ventral nerve cord elongation, brain reformation, and segmental ganglion appearance by 10 dpa [29]. Earthworm tail regeneration occurs through a temporal process similar to head regeneration. In *Eisenia andrei*, blastema forms beneath the wounded dermis at 1–3 dpa, and segmentation occurs within 7 dpa, when redifferentiation is not yet dynamic [30,31]. The qPCR analysis of the temporal expression of *Pex-ADAR* mRNA indicated that its transcription was inactivated in the early stages (1–7 dpa) when blastemal cells proliferate and the central nerve cord is reconstructed. Expression then suddenly rebounded at subsequent stages (10 dpa) when diverse cell types or tissues were regenerated in each segment. This suggests that ADAR activity is unrelated to the molecular mechanisms of dedifferentiation and is probably implicated in the generation of molecular diversity required for the re-specification of various cell types during redifferentiation [32].

In intact earthworms, *Pex-ADAR* mRNA expression was mainly observed in gut epithelia, the ventral nerve cord, and the dorsal vessel. Similarly, the ventral nerve cord of *C. elegans* showed intensive expression of *adr-1*, whose function is pertinent to normal chemotaxis behavior [27]. In addition, in mammals, RNA editing by ADAR1 in epithelial cells is essential for maintaining tissue homeostasis [33]. During the period of active redifferentiation, *Pex-ADAR* mRNA expression was mainly observed in the regenerating muscle layers and related connective tissues, suggesting the possibility that the RNA-editing enzyme *Pex-ADAR* is involved in muscle redifferentiation during earthworm regeneration. During mouse development, ADAR1 shows tissue-specific roles in skeletal myogenesis, and ectopic expression of ADAR1 retards the myotube fusion of myoblasts and muscle development. It is thought that ADAR1 expression displays programmed alteration that is coordinated with differentiation cues [34].

## 5. Conclusions

Taken together, earthworm *Pex-ADAR*, belonging to the ADAR1 family, is probably linked to the re-specification of muscle cells and tissues. The RNA editing activity of the enzyme may contribute to the generation of molecular diversity, which is required for muscle redifferentiation. Our subsequent research will focus on uncovering the molecular mechanisms behind how *Pex-ADAR* activity contributes to muscle cell re-specification from blastemal cells.

## Supplementary Materials

The following are available online https://www.mdpi.com/2079-7737/9/12/448/s1: Figure S1. Phylogenetic relationships among ADAR1s based on the NJ method. Phylogenetic analysis indicated that earthworm *Pex-ADAR* can be clustered with ADAR1s from other metazoans. ADAR2s and ADAR3s were grouped separately from ADAR1. The numbers at the nodes are scores from 1000 bootstrap re-samplings of the data, and unlabeled nodes are supported by fewer than 500 bootstraps; Figure S2. The micrographs provided in Figure 1A,A’ (Figure S2A) and Figure 5C,C’ (Figure S2B) with nuclei visualized with DAPI (×40, scale bar: 650 µm; ×200, scale bar 125 µm). A’ and B’ of Figure S2A represent the higher-magnification views of the corresponding white boxes in A and B.
