# Supplementary material for: Identification and Spatiotemporal Expression of Adenosine Deaminases Acting on RNA (ADAR) during Earthworm Regeneration: Its Possible Implication in Muscle Redifferentiation
Source: Biology (Basel). 2020 Dec 5;9(12):448. doi: 10.3390/biology9120448 (PMC7762157; doi:10.3390/biology9120448)
Supplement: Supplementary file 1 [file biology-09-00448-s001.zip › biology-990401-supplementary.pptx]

## Slide 1
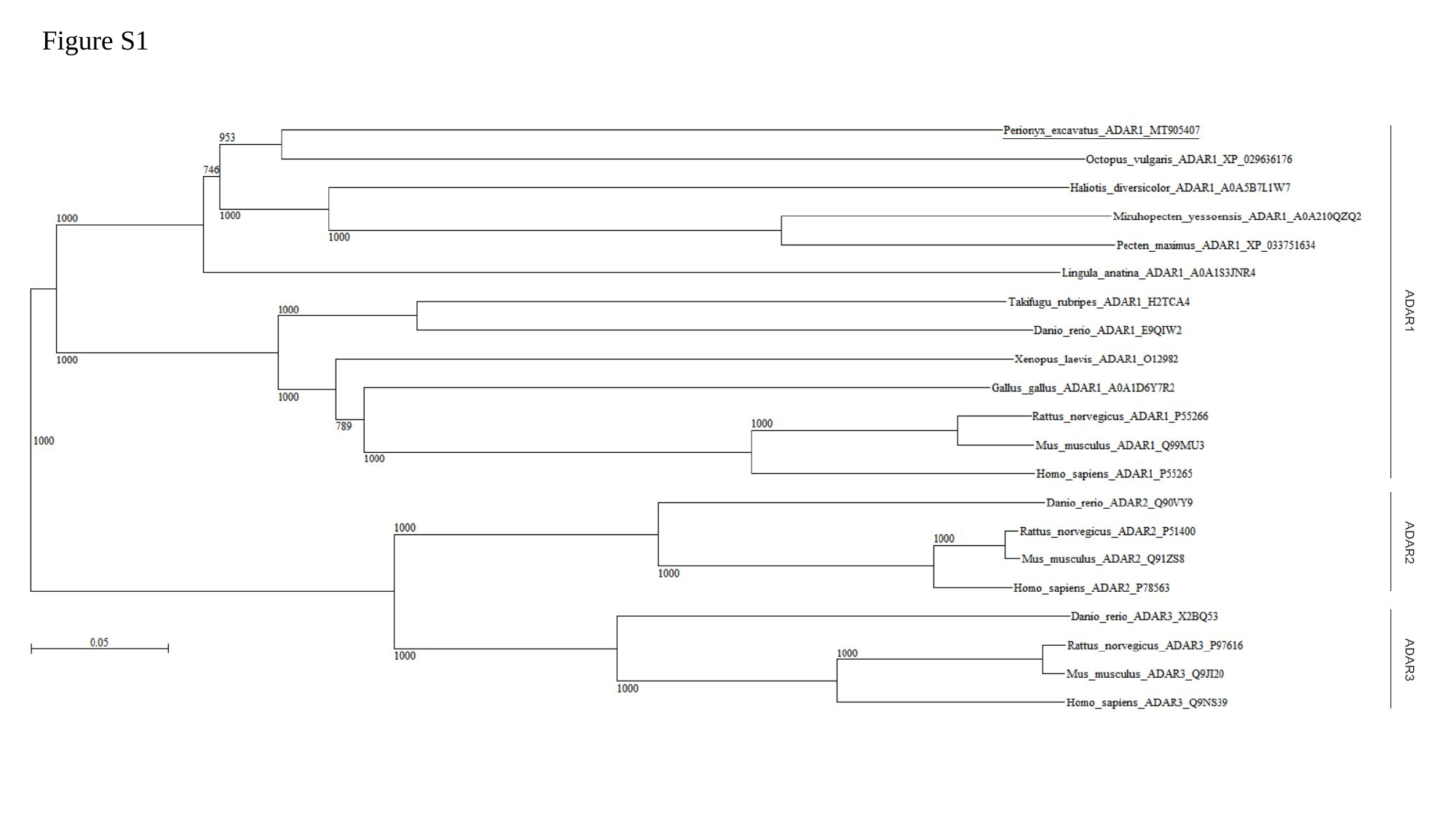

Figure S1

## Slide 2
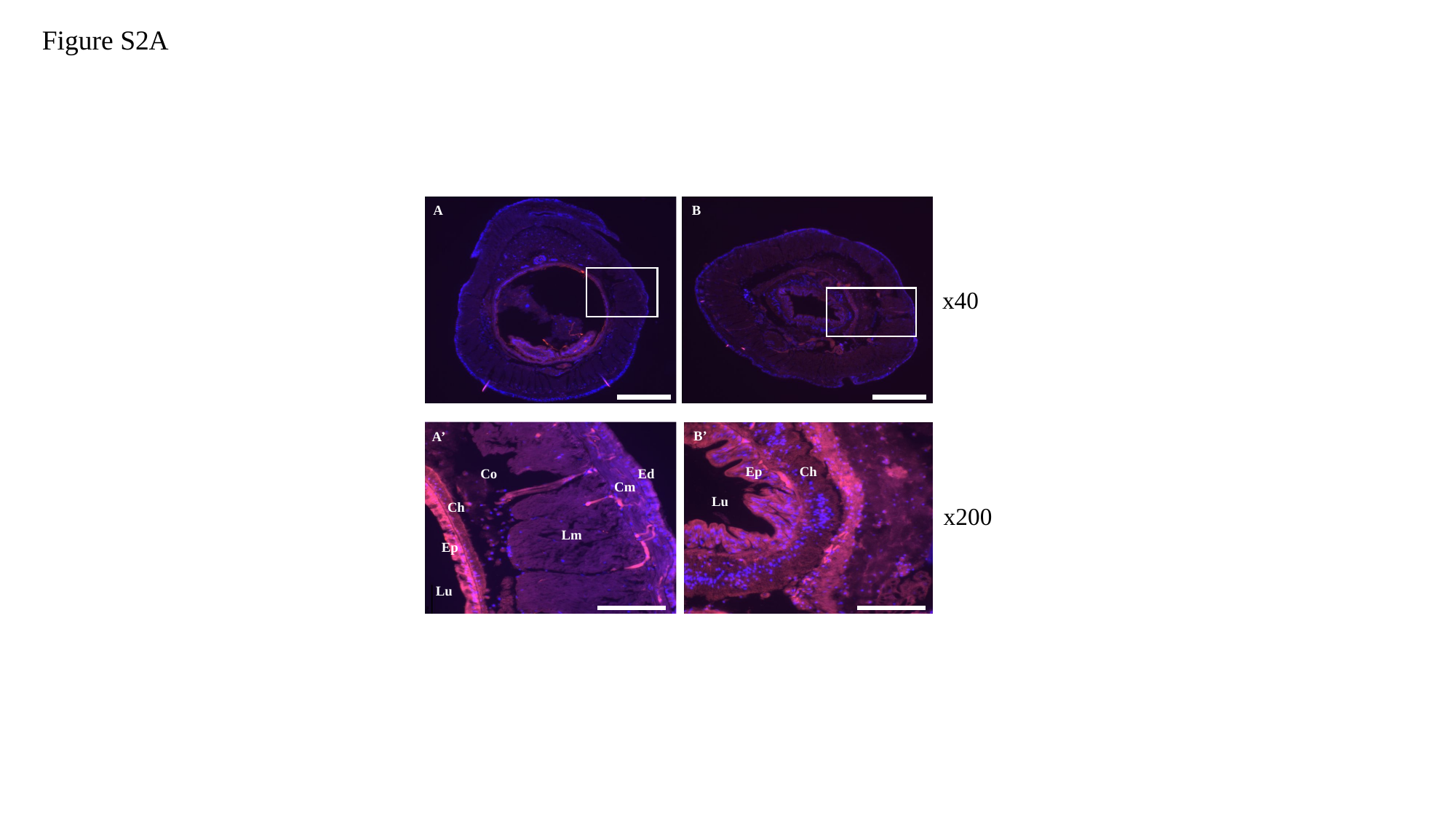

Figure S2A
A
Ep
Lu
Lu
Ch
Ep
Co
Ed
Cm
Lu
Ch
Lm
B
B’
A’
x40
x200

## Slide 3
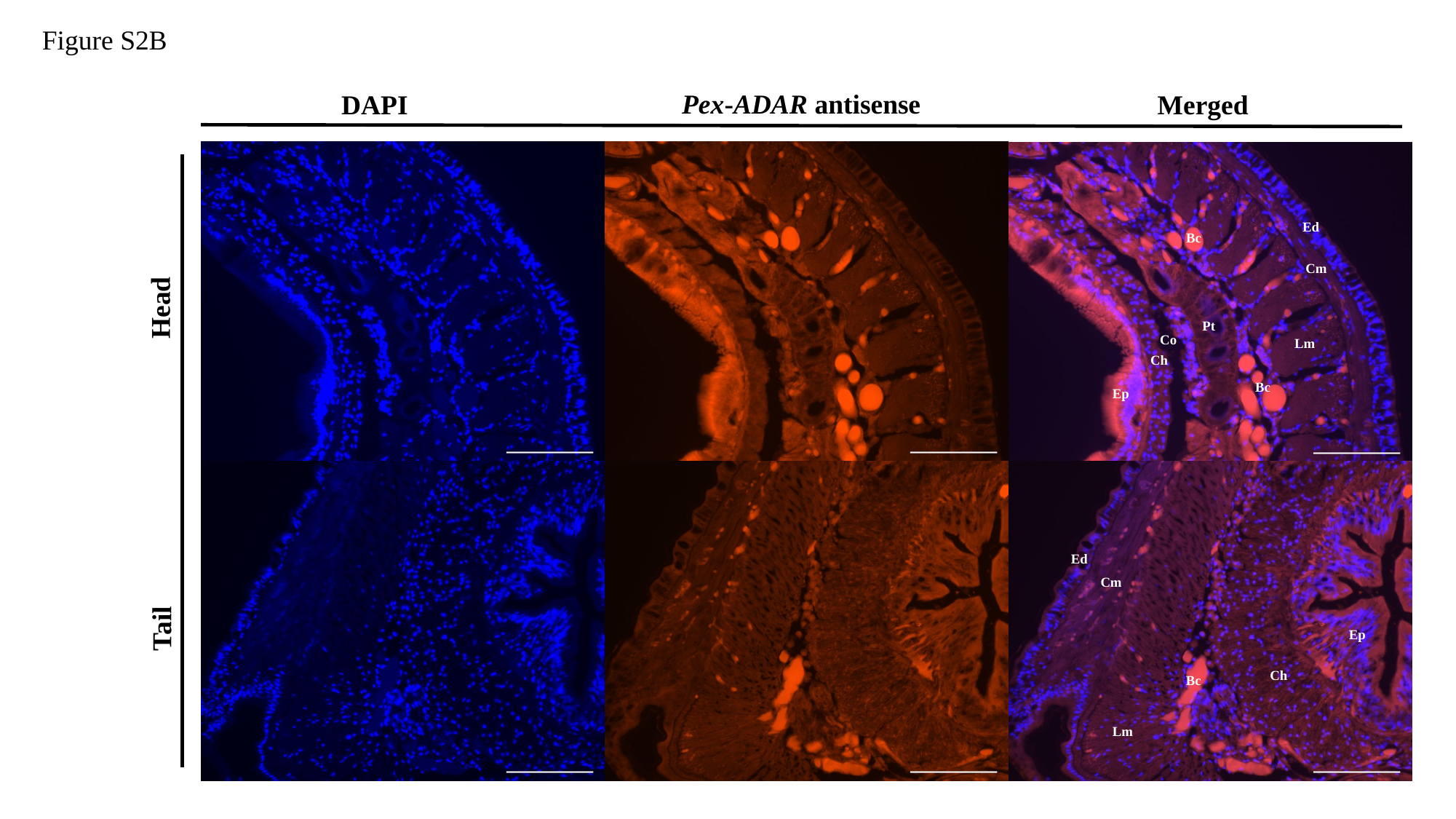

Figure S2B
Pex-ADAR antisense
Merged
DAPI
Ed
Bc
Cm
Head
Pt
Co
Lm
Ch
Bc
Ep
Ed
Cm
Tail
Ep
Ch
Bc
Lm
